# Agreement and repeatability of scotopic pupil size measurement with the 2WIN-S portable refractor in Chinese adults

**DOI:** 10.1038/s41598-024-66540-w

**Published:** 2024-07-08

**Authors:** Yibing Zhou, Xingru He, Ziming Liu, Ling Xu, Liangzhe Li, Jiayan Chen, Jiahui Zhao, Ruyi Li, Chunhong Yan, Cui Yu, Fei Yu, Wei He, Guanghao Qin, Sile Yu

**Affiliations:** 1 School of Public Health, Dalian Medical University, Dalian, China; 2 Department of Clinical Research, He Eye Specialist Hospital, Shenyang, China; 3 School of Public Health, He University, Shenyang, China; 4 School of Optometry, He University, Shenyang, China

**Keywords:** Medical research, Outcomes research

## Abstract

To assess the agreement and repeatability of scotopic pupil size measurement using 2WIN-S (Adaptica, Padova, Italy) portable refractor in Chinese adults. This prospective non-randomized open-label controlled study assessed the scotopic pupil size of 100 right eyes using OPD-Scan III (Optical path difference) (Nidek Technologies, Gamagori, Japan) and 2WIN-S. OPD-Scan III and 2WIN-S measure pupil size using infrared light and detector, while 2WIN-S measures bilateral eyes simultaneously, OPD-Scan III measures unilateral eyes individually. Participants were first measured once using OPD-Scan III and two consecutive measurements were performed using 2WIN-S after 15 min of rest interval. The primary outcome was to evaluate the agreement between 2WIN-S and OPD-Scan III, and the secondary outcome was to evaluate the repeatability of 2WIN-S. Scotopic pupil size of 100 right eyes of 100 adults (28 male and 72 female) aged 18–53 years (mean 36 ± 12 years) was assessed using OPD-Scan III and 2WIN-S, respectively. The mean scotopic pupil size of OPD-Scan III and 2WIN-S was recorded to be 6.24 ± 0.88 mm and 6.27 ± 0.81 mm, respectively. For the mean scotopic pupil size of OPD-Scan III and 2WIN-S the difference was − 0.03 mm (95%CI − 0.10 to 0.04 mm), *p* = 0.445, the 95% limits of agreement (LOA) was − 0.71 to 0.66 mm. ICC between the two devices was 0.92 (95% CI 0.88–0.94) (ICC > 0.9 indicates excellent consistency). Coefficients of repeatability (CoR) of 2WIN-S was 0.37, which has a high repeatability. For the mean scotopic pupil size of 2WIN-S of the repeated measurements, the difference was -0.04 mm (95%CI − 0.08 to 0.01 mm), *p* = 0.019, the 95% limits of agreement (LOA) was − 0.41 to 0.32 mm, with a narrow LOA. However, the majority of the variations were less than ± 0.50 mm (98% of scotopic pupil size measurements were below this threshold), within the clinically acceptable range (± 0.50 mm). Our study showed excellent agreement between 2WIN-S and OPD-Scan III (ICC > 0.9) and a good repeatability of 2WIN-S (CoR = 0.37). This study suggests a novel technique for measuring pupillary responses in low light conditions, which can be considered an alternative to OPD-Scan III in clinical settings.

## Introduction

Pupil size is controlled by the autonomic nervous system and primarily changes in response to light stimulation on the retina, and can be modulated by factors such as cognitive activity and accommodation^1^.Pupil size has a significant impact on visual function and performance in an individual, and therefore, pupil parameters such as scotopic pupil size have gained attention from clinicians as a vital parameter for postoperative refractive surgery outcomes^2,3^. The relevance of scotopic pupil size on postoperative quality of vision has been documented and researched in refractive surgery studies^4–6^. Therefore, it is necessary to accurately measure scotopic pupil size in preoperative patients.

While current technology allows us to measure scotopic pupil size in low-light illumination (< 0.05 lx)^7^. Historically, the measurement of pupil size included the use of a pupil ruler, which is known for its drawback of being very subjective and lacking precision. While infrared pupillometry devices such as OPD-Scan III (Optical path difference) (Nidek Technologies, Gamagori, Japan) and Sirius (Schwind Eye-Tech-Solution, Kleinostheim, Germany) are available for accurately and reliably measuring pupil size^8,9^, Nevertheless, the aforementioned equipments are costly and cumbersome to move in terms of screening and field trial logistics settings^4^. The OPD-Scan III (Nidek Technologies, Gamagori, Japan) is an auto keratometer, corneal topographer, autorefractor, aberrometer, and pupillometer/pupillographer workstation. Multiple studies have demonstrated the accuracy of OPD-Scan III in measuring pupil size^10–13^.

The 2WIN-S (Adaptica, Padova, Italy) is a compact and effective autorefractor with an integrated infrared pupillometer for capturing a scotopic pupil picture. The 2WIN-S incorporates a photo refractometer with an occlusion tube stabilising the focus distance and brightness. Additionally, it functions as a darkroom, enabling the examination to be conducted under any lighting conditions^14,15^. Previous studies have validated 2WIN-S as a viable tool for the detection of refractive errors such as myopia or amblyogenic risk factors such as significant hyperopia and/or anisometropia^15–18^. However, the agreement and repeatability of the 2WIN-S device for pupil size measurements in adults has not been reported in the literature. This study assessed the agreement and reproducibility of scotopic pupil size measurements using 2WIN-S compared with OPD-Scan III in Chinese adults. In addition, repeated measurements of the 2WIN-S were also evaluated.

## Results

**Figure 1:**
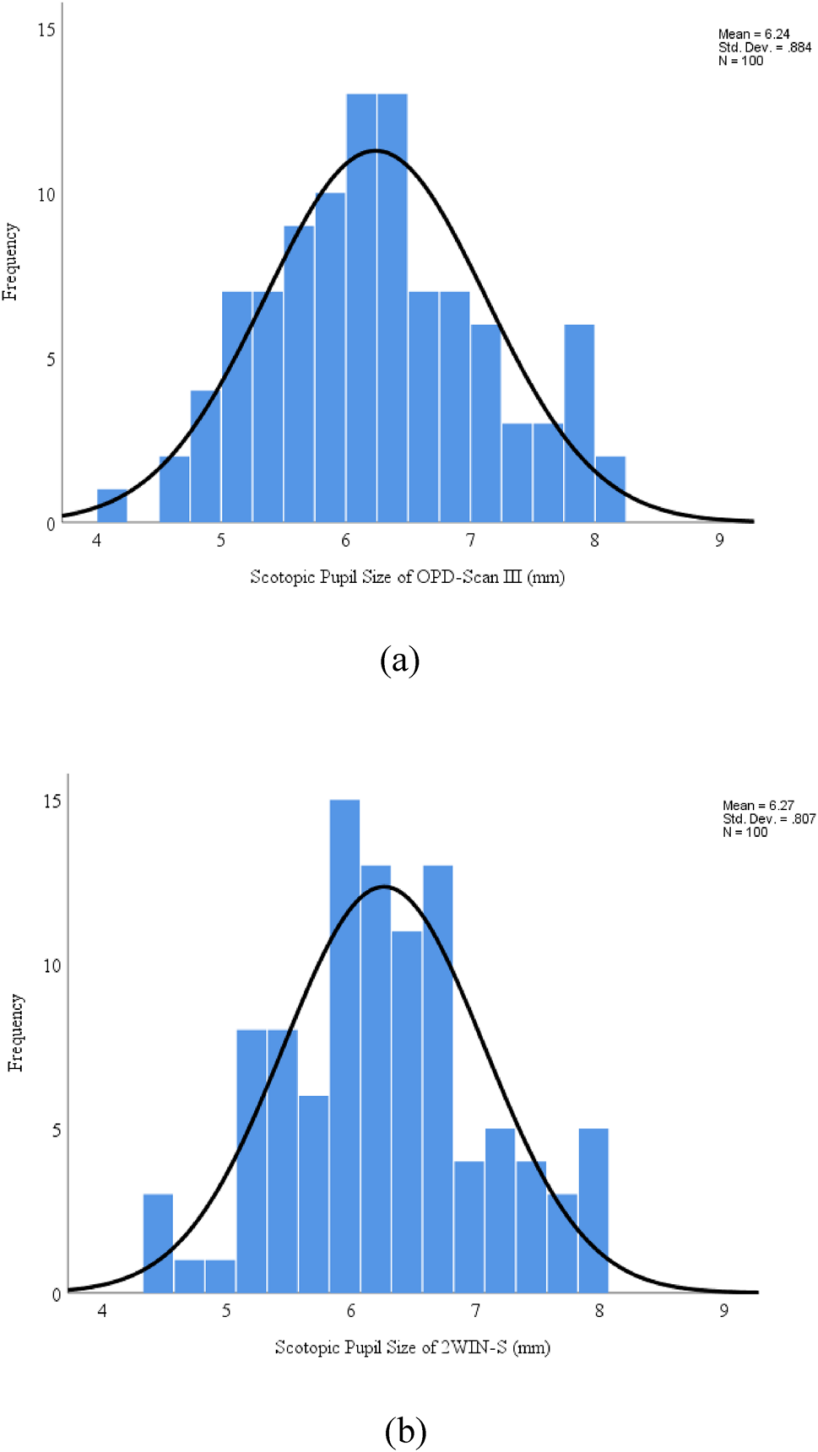
(**a**) Frequency distribution of scotopic pupil size with OPD-Scan III measurement. (**b**) Frequency distribution of scotopic pupil size with 2WIN-S measurement. OPD-Scan III: Optical path difference-Scan III.

The final analysis included 100 right eyes of 100 subjects (28 males and 72 females, 36 ± 12 years). The data adheres to a normal distribution, as determined by the Kolmogorov–Smirnov test. Table 1 provides the agreement of scotopic pupil size acquired with OPD-Scan III and 2WIN-S. Table 2 provides the repeatability of 2WIN-S. Figure 1a, b show the distribution of scotopic pupil sizes for OPD-Scan III and 2WIN-S.

**Table 1:** Comparison of measurements of scotopic pupil size between OPD-Scan III and 2WIN-S.

Pupil Size	OPD-Scan III	2WIN-S	Paired Mean Difference (95%CI)	*P* value (paired t-test) Δ		ICC (95%CI)
	Mean ± SD	Mean ± SD		*t*	*p*	
Scotopic (mm)	6.24 ± 0.88	6.27 ± 0.81	− 0.03 (− 0.10 to 0.04)	− 0.768	0.445	0.92 (0.88 to 0.94)

CI, confidence interval; SD, standard deviation; mm, Millimeter; Δ Comparison between OPD-Scan III and 2WIN-S (OPD-Scan III reading minus 2WIN-S reading); ICC, the intraclass correlation coefficient; OPD-Scan III: Optical path difference-Scan III.

**Table 2:** Repeatability of 2WIN-S.

Pupil Size	M1	M2	Paired Mean Difference (95%CI)	*P* value		CoR (95%CI)
	Mean ± SD	Mean ± SD		*t*	*p*	
Scotopic (mm)	6.24 ± 0.79	6.29 ± 0.83	− 0.04 (− 0.08 to 0.01)	− 2.379	0.019	0.37 (0.32 to 0.43)

M1, the mean value of the first measurement of 2WIN-S; M2, the mean value of the first measurement of 2WIN-S; mm, Millimeter; CoR, coefficients of repeatability; OPD-Scan III: Optical path difference-Scan III.

### Scotopic assessment

Table 1 provides descriptive data for the measurement of scotopic pupil size using different devices, with the mean OPD-Scan III and 2WIN-S being 6.24 ± 0.88 mm and 6.27 ± 0.81 mm, respectively.

### Agreement

**Figure 2:**
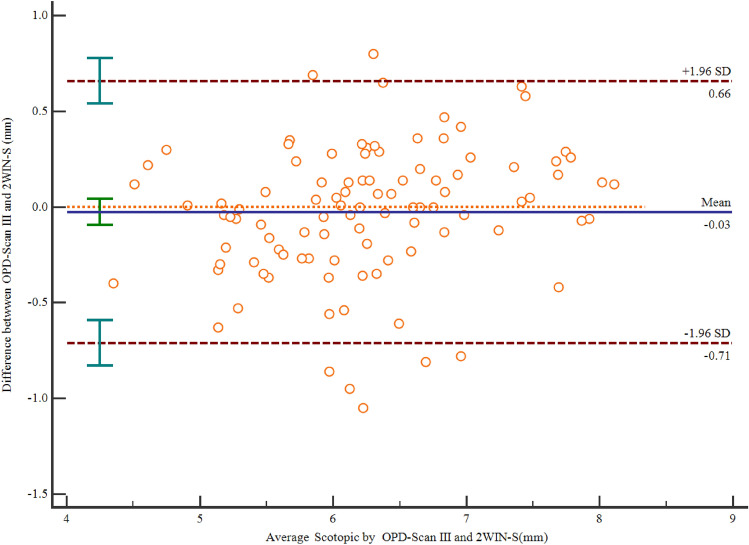
Bland–Altman plot showing the bias and 95% limit of agreement of OPD-Scan III compared to 2WIN-S for scotopic pupil size (A solid blue line indicates the mean bias and a dashed red line indicates the 95% bounds of agreement.). OPD-Scan III: Optical path difference-Scan III.

The mean difference for scotopic pupil size was -0.03 mm (with 85% of the differences within ± 0.50 mm), 95% LOA -0.71 to 0.66 m (Fig. 2). The scotopic pupil size did not show a significant difference between the measurement of the OPD-Scan III and 2WIN-S of the same individuals (*p* = 0.445) (Table 1). ICC between the two devices was 0.92 (95% CI 0.88 to 0.94).

### Repeatability

**Figure 3:**
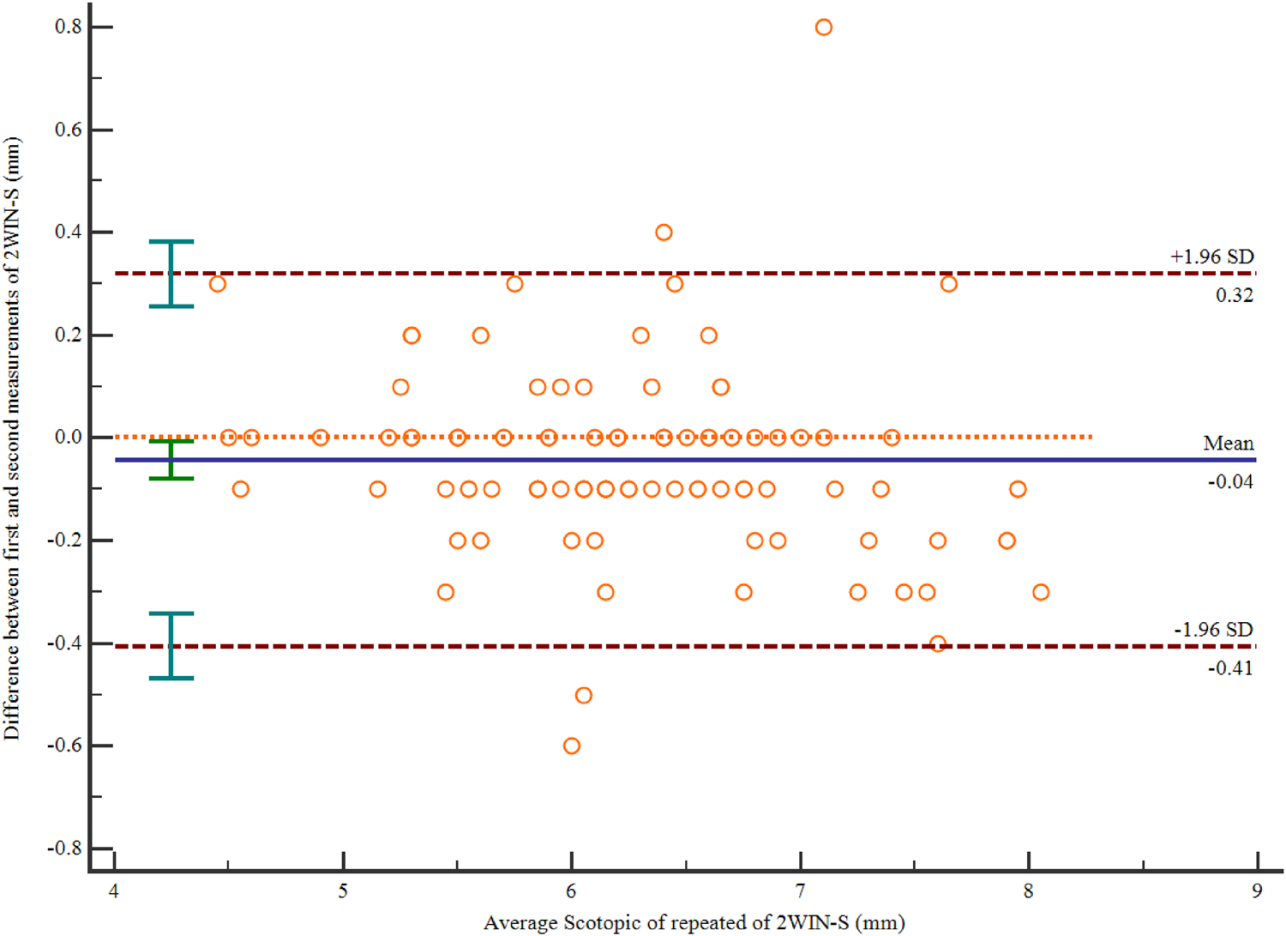
Inter-rater repeatability for 2WIN-S. Note the narrow range of differences between the repeated measurement (A solid blue line indicates the mean bias, and a dashed red line indicates the 95% bounds of agreement.). OPD-Scan III: Optical path difference-Scan III.

Table 2 provides the repeatability of scotopic pupil size measurements by 2WIN-S. The first measurement using 2WIN-S is 6.24 ± 0.79 mm and the second is 6.29 ± 0.83 mm. There was a significant difference between the first and second measurements using 2WIN-S (mean paired difference of − 0.04 mm, 95% LOA -0.41 to 0.32 mm, *p* = 0.019) (Fig. 3), and the majority of the variations were less than ± 0.50 mm (98% of scotopic pupil size measurements were below this threshold). The CoR of 2WIN-S was 0.37 mm (Table 2).

## Discussion

The contribution of pupil size to quality of vision disturbances in dim light environments in patients after corneal refractive surgeries has received much scrutiny^4,19,20^. Therefore, preoperative pupillometry has become one of the crucial parameters of surgery. Firstly, this study reported the scotopic pupil size measurement using 2WIN-S and compared it with OPD-Scan III in Chinese adults. The ICC value in our current study was 0.92 (with excellent agreement), since the agreement is > 90%, our findings suggest that the error caused by the device in clinical practice would be within a minimal acceptable range. The 85% difference between 2WIN-S and OPD-Scan III in this study was within ± 0.50 mm, suggesting that 2WIN-S has a good consistency with OPD-Scan III. Although there are statistical differences between 2WIN-S repeated measures, it is not comprehensive enough to look at whether there is a difference between means, so it also needs to be discussed in combination with B-A plots and clinically acceptable ranges (differences within ± 0.50 mm), most of which do not exceed ± 0.50 mm (98% of repeated measures have differences below this value). The CoR values of 2WIN-S (CoR 0.37 mm) surpass Colvard (CoR 1.16 mm/1.12 mm) and Procyon (CoR 0.64 mm/0.56 mm) infrared pupillometer which were reported in the previous studies^21,22^, signifying its notable high repeatability in scotopic pupil measurements. Therefore, combining the Bland–Altman plot and CoR values, we believe that 2WIN-S has good repeatability. Devices such as Colvard pupillometer are characterized by significant subjectivity and limited reproducibility, which are their main drawbacks. While the Procyon pupillometer has the capacity to measure both eyes at the same time, but at a considerable monitory cost^23^.

Both the 2WIN-S and OPD-Scan III utilize the infrared light principle for pupil measurement, prior research has confirmed that the OPD-Scan III is a highly reliable and appropriate clinical device for measuring pupil size^12,24^. Furthermore, these instruments can function under dim light conditions without interfering with the patient’s mydriasis. The primary drawback of the OPD-Scan III is its restriction to unilateral measurements and an extended measurement duration. The size of pupils in humans is regulated by the autonomic nervous system^25^. Thus monocular measurements may result in an enlarged pupil diameter in the unmeasured eye, elevating the risk of post-refractive glare and halos^26^. In contrast, 2WIN-S mitigates this risk by conducting binocular measurements. Our study results showed that 2WIN-S have a good agreement compared with OPD-Scan III on scotopic pupil size measurement. It is important to note that 2WIN-S operates independently of ambient light, creating a darkened environment with its occlusive tube. By configuring luminous parameters, it can effectively measure scotopic pupil size. Despite this limitation, 2WIN-S as a scotopic pupil measuring instrument is efficient, portable, and economical compared with other devices.

The present study exhibits certain limitations. It is limited by its focus on Chinese adults, without conducting tests on adolescents or other races. Iris colour affects pupil size measurements as confirmed by Schnitzler et al^27^. further study is needed to validate the findings among various races and ethnic groups with iris colour variations. Additionally, a comparison of photopic pupil size between the two machines was not performed due to substantial differences in the lumen output of their stimulating lights. Future research should consider comparisons with other pupillometers, such as Colvard, NeurOptics, and Procyon, to enhance the comprehensiveness of the findings. Nevertheless, our study provides a new reliable method for scotopic pupillometry to reference clinical practice. Ultimately, after examining the scotopic pupil size measurements acquired from both devices, we can confidently assert that the 2WIN-S has remarkable reliability and uniformity. Our current research is the first to employ 2WIN-S in assessing the repeatability and consistency of scotopic pupil measures in adults, further testing and reporting are needed to have a more comprehensive finding that can be generalize to a larger population.

## Methods

### Study design

This prospective non-randomized open-label controlled study was approved by the Ethics Committee of He Eye Specialist Hospital (IRB (2023) K006.01) and conducted by the Department of Clinical Research. The study was registered at ClinicalTrails.gov (NCT05717244), on January 29, 2023. Informed and signed consent was obtained from all participants of this study. This study was administered following the Declaration of Helsinki and the Clinical Trials Act.

### Participants

The study participants comprised Chinese adults aged 18 years and older, exhibiting no notable ocular health issues. Participants were recruited from He Eye Specialist Hospital between September and December 2023. Exclusion criteria were ocular pathology known to interfere with pupillometer outcome and pupil abnormalities such as glaucoma, iritis, cataract, corneal scarring, any history of ocular surgery, and history of trauma.

Piñero et al^28^. showed no difference between the left and right eyes, McAlinden et al^29^. showed possible correlation between the left and right eyes (except in asymmetric disease, such as keratitis), so in normal eyes, only one eye from each participant was used. Furthermore, if eyeball counts rather than participant numbers are used in the calculations, paired data may artificially narrow confidence intervals around the limits of agreement^29^. The right eye was used in this study because the right eye is the dominant eye in most people. Every subject received two binocular measurements in the 2WIN-S and one right eye measurement in the OPD-Scan III.

In this study, G*Power 3.1 software was used to calculate the sample size of the paired *t*-test. Assuming that the effect size of the two devices was 0.5^30^ and the alpha error was 5%, at least 44 people were needed to achieve the effect of 90% power. At the same time, McAlinden et al^29^. proposed that the sample size should be no less than 100 people to evaluate the agreement between the two methods. Increasing the sample size can accurately evaluate the degree of agreement and provide a stricter agreement interval.

### Examination procedures

The ocular evaluation was performed with a slit lamp (Takagi SM-70N, Kyushu Island, Japan) before the measurement. Scotopic pupil sizes were measured using the OPD-Scan III (Nidek Technologies, Gamagori, Japan) and 2WIN-S (Adaptica, Padova, Italy) in the same closed room. Prior to every measurement, the apparatus was adjusted, and the participant was directed to stay seated at the device for two minutes while the examination room lights were turned off (0.026 lx). A handheld illuminometer light meter (Konica Minolta LS-100, Tokyo, Japan) was utilized to confirm the illumination before measurement for every subject in order to guarantee constant illumination in the surroundings for every measurement. All measurements were performed by the same trained ophthalmologist (ZML).

**Figure 4:**
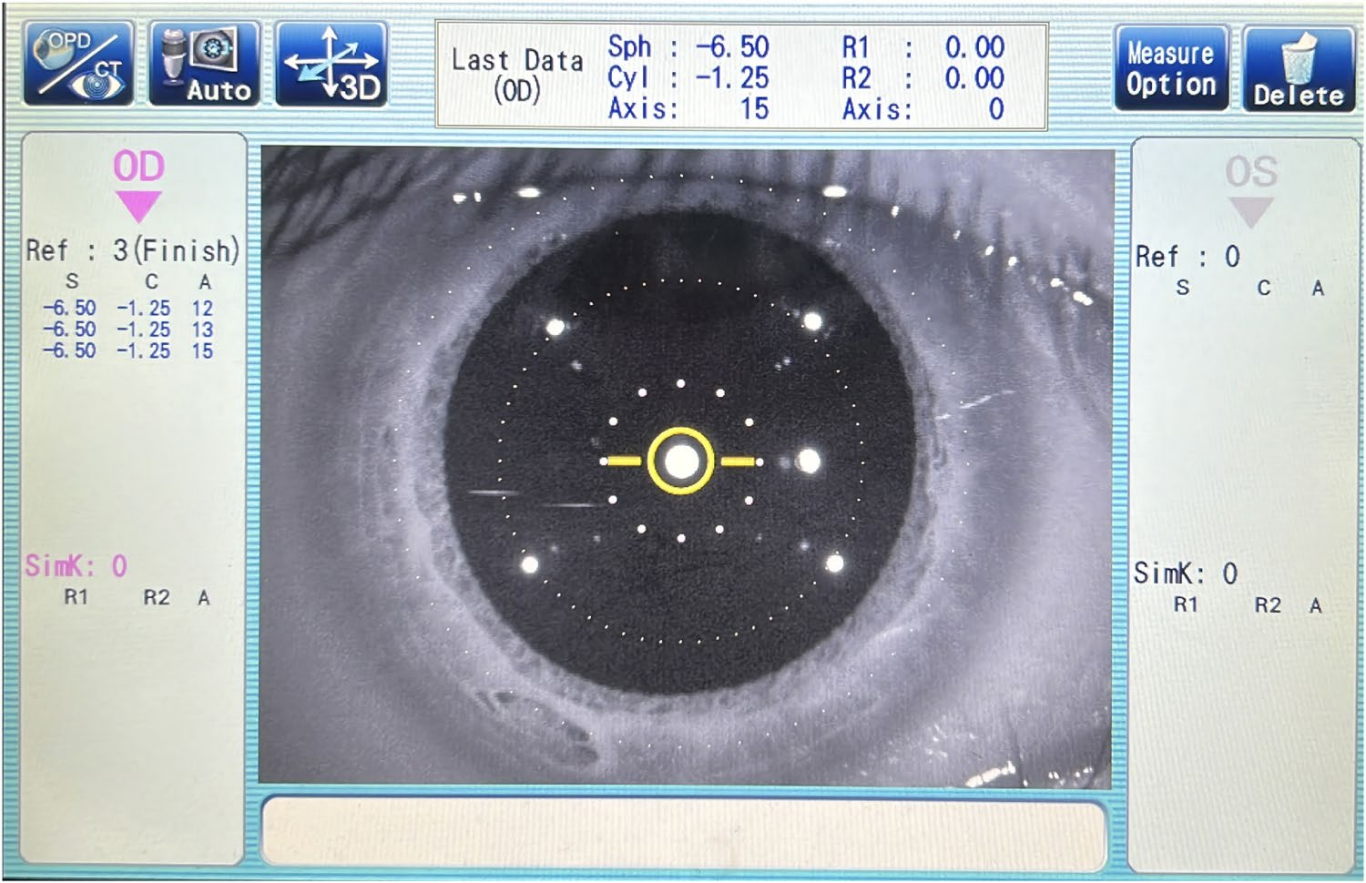
Pupil images of participants taken by OPD-Scan III. OPD-Scan III: Optical path difference-Scan III.

First, participants were asked to sit in the OPD-Scan III device with the left eye closed and measurements were taken with the right eye. The height of the table and jaw rest was set before adjustment. The participant was instructed to position their head (for each participant) with their jaw on the jaw rest and their forehead against the forehead rest, and to focus the window image by adjusting the lever. Once in measurement mode, the participant was instructed to focus his or her eyelid-lids as wide as possible on the object inside the device (a balloon) (0.042 lx). The examiner manipulated the handle of the OPD-Scan III instrument so that the cross point on the screen was located at the center of the pupil and adjusted the focus to make the image on the display clear, and then pressed the button to confirm the capture of the image. For blinks and images where the pupil was not adequately exposed, remeasurements were performed. A schematic of pupillometry is shown in Fig. 4.

**Figure 5:**
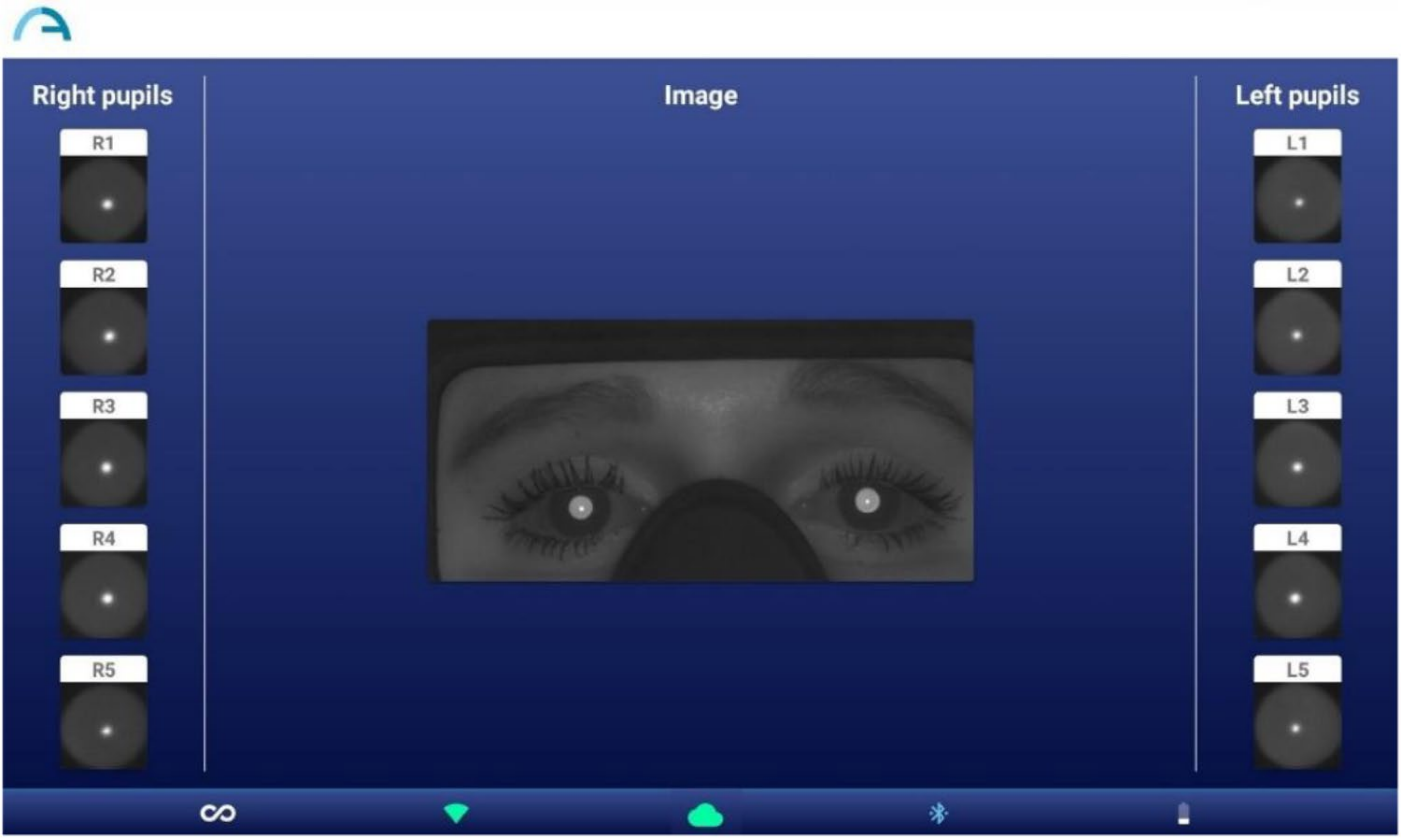
Pupil images of participants taken by 2WIN-S.

The same examiner conducted another set of 2WIN-S readings 15 min later under identical examination settings. The examiner pressed and held the ON/OFF button for one second to turn on the 2WIN-S. A beep indicates that 2WIN-S is powered on. The participants were asked to stand in front of the 2WIN-S device without glasses. Instead of placing the chamber's viewing portion against their eyes, we instructed the individuals to position it against their eyebrow. This made it possible to precisely line the participants’ eyes with the chamber and the refractometer within. From the home screen of the KALEIDOS App (Adaptica, Padova, Italy), we chose “Start a new measurement.” After the participant’s name and date of birth were typed and recorded. Next, the measurement type was set to the right eye and the start button was activated. A progress indicator appeared as soon as the measurement was initiated. This process required a minimum duration of three seconds. A solitary beep indicated the conclusion of the measurement, whereas a dual beep indicated precise processing of the data. The fixed target was achieved by looking at the bottom of the 2WIN-S occlusion tube (0.01 lx), asking participants to keep their eyes wide open and not blink, and asking participants to stare at the three white points at the bottom of the instrument during the measurement process, and not to follow the rotation of the three red lines when they appear, taking the best image that meets the requirements (Face directly and expose all pupils) (Fig. 5). A single beep signifies the completion of the measurement, while a double beep implies accurate processing and capture of the pupillary data. Finally, the pupil size was displayed on the KALEIDOS App (Adaptica, Padova, Italy). To assess the repeatability of the 2WIN-S, two consecutive scotopic pupil size were taken for each subject in the study. The right scotopic pupil size of all participants was included in the analysis.

### Statistical analysis

Data were analyzed using SPSS statistics software (version 25.0; SPSS Inc., United States). The mean standard deviation (SD) was used to express descriptive statistics for continuous variables. The distribution of scotopic pupil size data was explored using Kolmogorov–Smirnov tests. The difference between the two sets was compared using the paired t-test. The intraclass correlation coefficient (ICC) was determined for scotopic pupil size for correlation analyses. ICC values less than 0.50 were interpreted as poor, values between 0.50 and 0.75 as moderate, values between 0.75 and 0.90 as good, and values greater than 0.90 as excellent agreement^31^. The agreement between the scotopic pupil size measurement methods was investigated via Bland–Altman analysis with 95% limits of agreement (LOA). The higher the degree of agreement, the narrower the 95% limits of agreement with a reduced point scattering and points that are comparatively closer to the mean bias line. Coefficients of repeatability (CoR) were calculated as 1.96 × SD of the differences. Bland–Altman graphs were plotted using MedCalc statistical software (version 9.0.4; MedCalc Software bvba, Ostend, Belgium). A *p-value* of < 0.05 was considered to show statistical significance. Agreement was deemed clinically acceptable when the difference between measurements from different sets was within 0.50 mm.

### Ethical approval

This study was approved by the Ethics Committee of He Eye Specialist Hospital (IRB (2023) K006.01) and conducted by the Department of Clinical Research.

## Data Availability

Anonymized datasets generated and analyzed during the current study will be made available on reasonable request by the corresponding author (Guanghao Qin, qinguanghao2020@163.com).
